# Fast interaction dynamics of G-quadruplex and RGG-rich peptides unveiled in zero-mode waveguides

**DOI:** 10.1093/nar/gkab1002

**Published:** 2021-11-17

**Authors:** Satyajit Patra, Jean-Benoît Claude, Jean-Valère Naubron, Jérome Wenger

**Affiliations:** Aix Marseille Univ, CNRS, Centrale Marseille, Institut Fresnel, 13013 Marseille, France; Aix Marseille Univ, CNRS, Centrale Marseille, Institut Fresnel, 13013 Marseille, France; Aix Marseille Univ, CNRS, Centrale Marseille, FSCM – Spectropole, 13013 Marseille, France; Aix Marseille Univ, CNRS, Centrale Marseille, Institut Fresnel, 13013 Marseille, France

## Abstract

G-quadruplexes (GQs), a non-canonical form of DNA, are receiving a huge interest as target sites for potential applications in antiviral and anticancer drug treatments. The biological functions of GQs can be controlled by specifically binding proteins known as GQs binding proteins. Some of the GQs binding proteins contain an arginine and glycine-rich sequence known as RGG peptide. Despite the important role of RGG, the GQs-RGG interaction remains poorly understood. By single molecule measurements, the interaction dynamics can be determined in principle. However, the RGG–GQs interaction occurs at micromolar concentrations, making conventional single-molecule experiments impossible with a diffraction-limited confocal microscope. Here, we use a 120 nm zero-mode waveguide (ZMW) nanoaperture to overcome the diffraction limit. The combination of dual-color fluorescence cross-correlation spectroscopy (FCCS) with FRET is used to unveil the interaction dynamics and measure the association and dissociation rates. Our data show that the RGG–GQs interaction is predominantly driven by electrostatics but that a specific affinity between the RGG sequence and the GQs structure is preserved. The single molecule approach at micromolar concentration is the key to improve our understanding of GQs function and develop its therapeutic applications by screening a large library of GQs-targeting peptides and proteins.

## Graphical Abstract

**Graphical Abstract:**
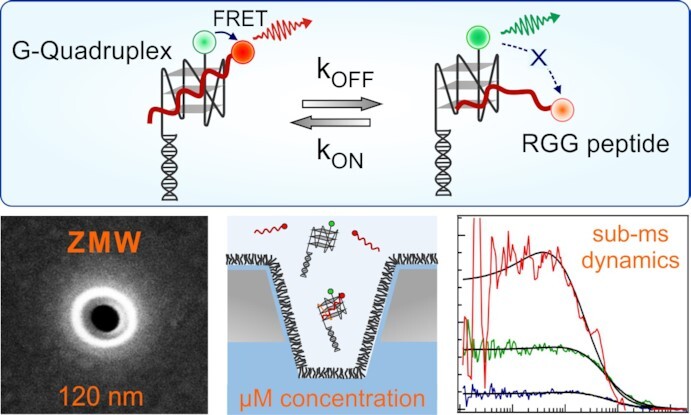
Nanoaperture to study fast dynamics between G-quadruplexes and peptides.

## INTRODUCTION

G-quadruplexes (GQs) are a non-canonical DNA structure formed through H-bonding and stacking interaction of guanine residues and further stabilized by monovalent cation, e.g. Na^+^ and K^+^(1–5). Genome-wide sequencing studies have revealed 700 000 potential GQs forming sequences particularly enriched in the promoter and telomere region of the human genome highlighting their importance in regulating key cellular processes such as transcription and replication (6,7). Furthermore, it has been also observed that the formation of stable GQ structure in the telomeric region of the chromosome can inhibit the activity of telomerase which is upregulated in 85% of the cancer cells (8,9). This has made GQs an interesting target site for potential anticancer therapy and has triggered a large number of biophysical studies focusing on GQs structure and interaction dynamics (10–22). The GQs function is regulated by some proteins, many of them feature an arginine and glycine rich motif known as RGG motif. In a recent report, Brázda *et al.* have compared the amino acid sequences of 77 GQs binding human proteins and have found that all these proteins share a common 20 amino acid long arginine and glycine rich RGG motif of sequence RGRGRGRGGGSGGSGGRGRG (23,24). This RGG motif has a very good similarity with the RGG motif normally found for GQs binding proteins such as FMR1 and DHX 36 (23). It is believed that this RGG motif plays a crucial role in the recognition of the GQs structures (25–33). Previous structural studies based on NMR and X-ray crystallography indicate that RGG predominantly binds to the junction between duplex and GQs and the presence of GQs forces a sharp turn of the RGG peptide (32,33). Huang *et al.* have revealed the importance of the internal arrangement of arginine and the presence of aromatic amino acids in the RGG motif peptide in recognizing the GQs DNA structures (25). Recent work of Masuzawa *et al.* has also shown that the phenylalanine in the RGG domain of nucleolin is responsible for the binding with GQs (27). While these studies provide crucial information on the nature and arrangement of the amino acids in the RGG required for the binding with GQs, the dynamics information is still lacking. Particularly, the association and dissociation rates of the GQ–RGG complex remain unknown.

Single molecule measurements can in principle provide detailed information about the interaction dynamics (34). However, isolating a single molecule in the confocal detection volume of a diffraction-limited microscope requires concentrations in the picomolar to nanomolar range (35,36). This concentration range is not compatible with the GQ–RGG interaction which has a dissociation constant in the μM range (25,32). Therefore, conventional single-molecule fluorescence techniques on confocal microscopes cannot be used to study the RGG–GQ interaction. This lack of a proper technique mainly explains why the dynamic information about this complex remained elusive so far.

To overcome the concentration limit for single molecule studies and monitor the RGG–GQ interaction dynamics at μM concentration, we use here a 120 nm aluminium nanoaperture known as zero-mode waveguide (ZMW), to reach an attoliter (10^–18L^) detection volume (36,37). Pulsed-interleaved dual-color fluorescence cross-correlation spectroscopy (PIE-FCCS) becomes possible with single molecule resolution at micromolar concentration in the ZMW. This allows monitoring the previously unexplored interaction dynamics between human telomeric GQs and RGG at physiological concentration. Our data resolve the association and dissociation rates and provide new mechanistic insights on this interaction. While many review papers have discussed the interest in ZMW nanoapertures for single-molecule biophysics at high concentrations (36,38–43), real biophysical applications of ZMWs apart from DNA sequencing (44–46) remain quite scarce (47–52). This situation further emphasizes the need for a detailed experimental report of the application of ZMW nanoapertures to a real biophysical question and the scientific assessment of their performance in relevant biological conditions.

## MATERIALS AND METHODS

### Materials

#### Zero-mode waveguide fabrication

The zero-mode waveguide nanoapertures are fabricated into a 100 nm thick aluminium film deposited on a glass coverslip by focused ion beam (FIB) milling. The aluminium deposition on a clean glass coverslip is carried out by electron beam evaporation (Bühler Syrus Pro 710). To achieve the optimum performance in our ZMW, the aluminium deposition is performed with a deposition rate of 10 nm/s at a chamber pressure of 5 × 10^–7^ mbar. The ZMW nanoapertures are then milled into the aluminium film using focused gallium ion beam. The parameter of the focused ion beam is set to 10 pA current and 30 kV voltage, and the resolution of the gallium ion beam is about 10 nm.

#### DNA samples

In the present study, we have used four DNA samples, Alexa 546 labelled G-quadruplex (GQ-A546), Cy3B labelled G-quadruplex (GQ-Cy3B), Alexa 546 labelled 51 base long DNA single strand (ssDNA-A546) and a 51 bp long DNA double strand (dsDNA-A546). The GQs DNA consists of a duplex stem and a G-rich single stranded overhang containing the human telomeric oligonucleotide sequence [(GGGTTA)_3_GGG], which at appropriate salt concentration folds to form the G-quadruplex (GQ). So, the GQ DNA consists of two strands, A GQ forming strand and a complementary strand. The GQ forming strand is labelled with either Alexa 546 or Cy3B while the complementary strand is non-labelled. The base sequences of the DNA are given below

#### GQ-DNA

##### Alexa 546 labelled GQ forming strand

5′-(Alexa 546) TTG GGT TAG GGT TAG GGT TAG GGT TTG GCT GCG CAG GAC GAG CGC-3′.

##### Cy3B labelled GQ forming strand

5′-(Cy3B) TTG GGT TAG GGT TAG GGT TAG GGT TTG GCT GCG CAG GAC GAG CGC-3′.

##### Complementary strand of the GQ-DNA

5′-GCG CTC GTC CTG CGC AGC CA-3′.

#### ssDNA-A546

5′ -CCT GAG CGT ACT GCA GGA TAG CCT ATC GCG TGT CAT ATG CTG T(Alexa546)TC AGT GCG-3′.

#### dsDNA-A546

##### Forward strand

5′-CCT GAG CGT ACT GCA GGA TAG CCT ATC GCG TGT CAT ATG CTG T(Alexa546)TC AGT GCG-3′.

##### Complementary reverse strand

5′ -CGC ACT GAA CAG CAT ATG ACA CGC GAT AGG CTA TCC TGC AGT ACG CTC AGG-3′.

Alexa 546 labelled GQ forming strand and non-labelled complementary strand of the GQ-DNA, 51b DNA ss, and 51 bp DNA ds are purchased from IBA Life Solution (Göttingen, Germany). The Cy3B labelled GQ forming strand is purchased from Integrated DNA Technologies (Leuven, Belgium). All the DNA sequences are high performance liquid chromatography (HPLC) purified by the manufacturer. To prepare the GQ-DNA and the 51 bp DNA ds, the corresponding forward and reverse complementary strands were annealed at 80 μM concentration in a buffer containing 10 mM Tris, 100 mM KCl at pH 7.5. The annealing is performed by first heating the equimolar mixture of the complementary strands at 95°C for five minutes followed by a slow, stepwise cooling to room temperature. The annealed DNA samples are diluted to a desired concentration in a 10 mM Tris, 100 mM KCl, 2% Tween 20, pH 7.5 buffer for the final measurements. For the measurements with high salt concentrations, the desired amount of KCl is added into the buffer.

#### RGG peptide

The arginine (R) and glycine (G) rich peptide RGG is purchased from JPT Peptide Technologies GmbH (Berlin, Germany). The amino acid sequence of the RGG peptide is RGRGRGRGGGSGGSGGRGRG-Cys (Alexa 647). An extra cysteine is added in the C-terminal end to label the peptide with Alexa 647 fluorophore. The RGG peptide is of high performance liquid chromatography purified grade. The manufacturer has reported 95.1% purity for this peptide (Supporting Information Supplementary Figure S1). The peptide is used as received without further purification.

#### Surface passivation of the zero-mode waveguide nanoapertures

In order to avoid unwanted nonspecific adsorption of the biomolecules on the surface of zero-mode waveguide (ZMW), the ZMW nanoapertures are passivated with a silane-modified methoxy polyethylene glycol of molecular weight 1000 Da (mPEG-silane 1000, Nanocs). The ZMW nanoapertures are first rinsed with water, ethanol and isopropanol and finally cleaned with air plasma for 5 min to remove any organic impurities. The cleaned ZMWs are then covered with a solution of 1 mg/ml mPEG-silane in 96% ethanol with 1% acetic acid and kept in an argon environment for 24 h. Finally, the ZMWs are rinsed with ethanol to remove any unadsorbed m-PEG-silane and dried by blowing synthetic air.

### Experimental methods

#### Dual color fluorescence correlation spectroscopy (FCCS) set up

Pulsed interleaved (PIE) dual color fluorescence cross correlation spectroscopy (FCCS) measurements are carried out in a home build confocal microscope. The dual color interleaved pulsed excitation is achieved by combining a iChrome TVIS laser (Toptica GmbH, pulse duration ∼ 3 ps) at 557 nm providing the green excitation and a 635 nm LDH series laser diode (PicoQuant GmbH, pulse duration 60 ps) providing the red excitation. Green and red laser pulses are alternated to achieve the pulsed interleaved excitation to separate the red excitation of the acceptor from the FRET excitation. This efficiently avoid cross-talk issues in FCCS. For this purpose, both the lasers are operated at 40 MHz repetition rate with constant 12.5 ns delay between them. The two laser beams are spatially overlapped by injecting them into a polarization-maintaining single mode optical fiber of 2 m length. The laser beam is then collimated by using a 10× objective lens (Nikkon Plan Fluor, NA 0.3) and coupled into a Nikon Eclipse Ti-U inverted microscope by a multiband dichroic mirror (ZT 405/488/561/640rpc, Chroma). In the microscope, a water immersion objective lens (Zeiss C-Apochromat 63× 1.2 NA) is used to focus the light on individual ZMW nanoapertures and to collect the fluorescence in an epifluorescence configuration. The collected fluorescence light is then guided through the same multiband dichroic mirror and an emission filter (ZET405/488/565/640mv2, Chroma) to block the laser back reflection and finally spectrally separated into two detection channel using a dichroic mirror (ZT633RDC, Chroma). Each detection channel is equipped with 50 μm pinhole and emission filters (donor fluorescence collection from 570 to 620 nm and acceptor fluorescence collection from 655 to 750 nm) for further spectral purification of the fluorescence signal. Two avalanche photodiodes (MPD series, <50 s time jitter, PicoQuant) are used to detect the green and red fluorescence signal. The photodiode signals are connected to a single photon counting module (HydraHarp 400, PicoQuant) and each fluorescence photon is recorded with individual timing and channel information in a time tagged time resolved (TTTR) mode.

The excitation power of the red laser is kept at 20 μW for all the measurements. The green laser intensity is 20 μW for the measurements with 1 and 5 μM GQs. For higher concentrations of GQs and other DNA sequence (i.e. 20 and 40 μM) the green laser power is reduced to 1 μW to avoid the saturation of the detectors. All the measurements are performed at 20°C temperature.

#### Fluorescence cross correlation and autocorrelation analysis

The fluorescence intensities in the green and red detection channel are correlated to generate the cross-correlation function. The general expression of the cross-correlation function for the two species i and j is given by

(1) $$\begin{equation*}{G_{ij}}\;\left( \tau \right) = \frac{{\langle \delta {F_i}\left( t \right)\delta {F_j}\left( {t + \tau } \right)\rangle }}{{\langle {F_i}\left( t \right)\rangle \langle {F_j}\left( t \right)\rangle }}\end{equation*}$$

where *δF*_i_(*t*) and *δF*_j_(*t+τ*) are the fluorescence intensity fluctuation of species *i* and *j* at time *t* and *t + τ*, respectively. When, *i* = *j*, i.e both the species are same then it is called autocorrelation function. Here, we are essentially dealing with a mixture of green only (G), red only (R) and green-red (GR) labelled molecules. The total concentration of green and red labelled species can be described as *C*_G,tot_ = *C*_G_ + *C*_GR_ and *C*_R,tot_ = *C*_R_ + *C*_GR_. Here, *C*_G_, *C*_R_ and *C*_GR_ are the concentrations of green only, red only and green-red labelled species. The cross-correlation function is sensitive to only dually labelled species GR, while green and red autocorrelation function is sensitive to the total concentration of green (*C*_G,tot_) and red labelled species (*C*_R,tot_) respectively. Therefore, under dual color excitation the expression of auto and cross-correlation function can be written as (53–55)

(2) $$\begin{equation*}{G_{GG}}\;\left( \tau \right) = \frac{{{C_G}{G_{diff,G}}\left( \tau \right) + {C_{GR}}{G_{diff,GR}}\left( \tau \right)}}{{{V_{eff}}C_{G,tot}^2}}\;\end{equation*}$$

(3) $$\begin{equation*}{G_{RR}}\;\left( \tau \right) = \frac{{{C_R}{G_{diff,R}}\left( \tau \right) + {C_{GR}}{G_{diff,GR}}\left( \tau \right)}}{{{V_{eff}}C_{R,tot}^2}}\;\end{equation*}$$

(4) $$\begin{equation*}{G_{GR}}\;\left( \tau \right) = \frac{{{C_{GR}}{G_{diff,GR}}\left( \tau \right)}}{{{V_{eff}}{C_{G,tot}}{C_{R,tot}}}}\;\end{equation*}$$

where *G*_diff,i = G,R,GR_ represents the diffusion related part of the correlation function and can be expressed mathematically as

(5) $$\begin{equation*}{G_{diff,i\; = \;G,R,\;GR}} = {\left( {1 + \frac{\tau }{{{\tau _{D,i}}}}} \right)^{ - 1}}\;{\left( {1 + \frac{\tau }{{{\kappa ^2}{\tau _{D,i}}}}} \right)^{ - \frac{1}{2}}}\end{equation*}$$

In the above expression, τ_D,i_ is the diffusion time of the molecule through the observation volume and κ is the ratio of the axial and transverse radius of the observation volume (*V*_eff_). The total correlation function can be expressed as

(6) $$\begin{equation*}{G_i} = {G_{trp,i}}\;{G_{fret,i}}{G_{diff,i}}\end{equation*}$$

where, ${G_{trp,i}}$ and ${G_{fret,i}}$ represents the correlation arising from the contribution of triplet state and energy transfer dynamics respectively. The crosscorrelation function is fitted according to the following equation

(7) $$\begin{eqnarray*}{G_{GR}}\;\left( \tau \right) &=& {G_{GR}}\;\left( 0 \right)\left[ {1 - Sexp\left( { - \frac{\tau }{{{\tau _S}}}} \right)} \right]\nonumber\\ &&\left[ {{{\left( {1 + \frac{\tau }{{{\tau _{D,i}}}}} \right)}^{ - 1}}{{\left( {1 + \frac{\tau }{{{\kappa ^2}{\tau _{D,i}}}}} \right)}^{ - \frac{1}{2}}}} \right]\end{eqnarray*}$$

where *S* and τ_S_ represent the amplitude and time scale of the interaction dynamics respectively and *G*_GR_(0) is the amplitude of the crosscorrelation function at lag time τ = 0. From equation 4, we can write ${G_{GR}}\;( 0 ) = \frac{{{C_{GR}}}}{{{V_{eff}}{C_{G,tot}}{C_{R,tot}}}}\;$. The cross correlation function does not contain any contribution from the triplet state because there would be no correlation between individual triplet state dynamics of the green and red fluorophores. Therefore, the crosscorrelation function is the product of the contribution from energy transfer dynamics and diffusion.

The interaction dynamics between GQs and RGG is leading to a FRET efficiency fluctuation resulting a pronounced anticorrelation between green and red fluorescence signal. This anticorrelation decreases the amplitude of the cross correlation and introduces a rise term in the crosscorrelation function in the earlier lag times (Supplementary Figure S2, Supporting Information) (56). This rise term represents the interaction dynamics of the GQ–RGG complex. Fitting this rise term with the exponential term of equation 7 provides the amplitude and time scale of the interaction dynamics.

The fluctuation of FRET efficiency due to interaction dynamics will be also leading to a fluctuation in the donor fluorescence intensity and will introduce a bunching term in the green autocorrelation function. Therefore, green autocorrelation function will be the product of all the three contributions i.e from triplet state, interaction and diffusion dynamics. The green autocorrelation curves are fitted into the following equation

(8) $$\begin{eqnarray*}{G_{GG}}\;\left( \tau \right) &=& {G_{GG}}\;\left( 0 \right)\left[ {1 + {A_T}exp\left( { - \frac{\tau }{{{\tau _T}}}} \right)} \right]\nonumber\\ && \left[ {1 + Sexp\left( { - \frac{\tau }{{{\tau _S}}}} \right)} \right]\nonumber\\ &&\left[ {{{\left( {1 + \frac{\tau }{{{\tau _{D,i}}}}} \right)}^{ - 1}}{{\left( {1 + \frac{\tau }{{{\kappa ^2}{\tau _{D,i}}}}} \right)}^{ - \frac{1}{2}}}} \right]\end{eqnarray*}$$

where *A*_T_ the amplitude of the triplet state dynamics, τ_T_ is the lifetime of the triplet state, and S and τ_S_ is the amplitude and relaxation kinetics of the interaction dynamics respectively. From the equation (2), we can derive the expression for the amplitude of green autocorrelation at lag time zero as ${G_{GG}}( 0 )\; = \frac{1}{{{V_{eff}}{C_{G,tot}}}}\;$.

#### Determination of bound fraction from cross-correlation and green autocorrelation analysis

The FCCS correlation amplitude does not only depend on the concentration of bound species featuring both green and red labels *C*_GR_, its denominator also contains the total concentration of green (*C*_G,tot_) and red labelled species (*C*_R,tot_), see Eq. (4). Here, the concentration of the red labelled species (RGG-A647) is fixed at 1 μM and the concentration of green labelled species (GQs-A546) is varied from 1 to 40 μM. We thus define the bound fraction as the fraction of the red-labelled species also featuring a green label out of the total red-labelled species. Therefore, we have calculated the bound fraction of RGG peptide by normalizing the crosscorrelation amplitude *G*_GR_(0) by the green autocorrelation amplitude *G*_GG_(0). So, from the expressions (2), (4), (7) and (8) we can write,

(9) $$\begin{equation*}{\rm{Bound\;fraction\;}} = \frac{{{{{G}}_{{\rm{GR}}}}\left( 0 \right)}}{{{{{G}}_{{\rm{GG}}}}\left( 0 \right)}}{\rm{\;}} = \frac{{{{{C}}_{{\rm{GR}}}}}}{{{{{C}}_{{\rm{R}},{\rm{tot}}}}}}\;.\end{equation*}$$

In the above expression, the ratio, *C*_GR_/*C*_R,tot_, quantifies the fraction of red labelled species (RGG-A647 in this case) bound to GQs. We plot this bound fraction as a function of GQs concentrations to determine the dissociation constant of GQ–RGG complex. A strength of the PIE-FCCS method is to be able to extract relevant information about the bound fraction despite the presence of a large excess of the green-labelled species respective to the red-labelled species (56,57).

#### Fitting model for the binding curve

The RGG bound fraction given by Eq. (9) is fitted as a function of the GQ concentration by a Hill equation model:

(10) $$\begin{equation*}{\rm{Bound\;fraction}}\; = \;\frac{1}{{1 + {K_{\rm D}}/\left[ {{\rm GQ}} \right]}},\end{equation*}$$

where *K*_D_ represents the dissociation constant (GQs concentrations at which 50% of the RGG is bound to GQs). The binding stoichiometry between GQs and RGG is 1:1 so that the Hill coefficient is set to 1 here.

#### Methods to determine the kinetics for the interaction dynamics between GQ-DNA and RGG peptide

Using FRET FCS approach we want to determine the interaction dynamics between GQs-DNA and RGG peptide. This binding and unbinding event is leading to a transition between a high FRET and a low FRET state of respective FRET efficiencies (*E*_1_) and (*E*_2_). Let us consider *k*_on_ and *k*_off_ is the association and dissociation rate constant for the GQ–RGG complex formation. The equilibrium constant for the transition from high FRET state to low FRET state can be expresses as the ratio of *k*_off_ and *k*_on_, i.e. *K* = *k*_off_/*k*_on_. At equilibrium, association rate is equal to dissociation rate, that means *k*_on_[GQ][RGG] = *k*_off_[GQ–RGG].

From this we can express the bound RGG fraction in terms of equilibrium constant *K* as

(11) $$\begin{eqnarray*} {\rm{Bound RGG fraction}}\, &=& \frac{{\left[ {GQ - RGG} \right]}}{{\left[ {RGG} \right] + \left[ {GQ - RGG} \right]}}{\rm{\;}}\nonumber\\ &=& \frac{{{k_{on}}\left[ {GQ} \right]}}{{{k_{on}}\left[ {GQ} \right] + {k_{off}}}}\, = \frac{{\left[ {GQ} \right]}}{{\left[ {GQ} \right] + K}}\nonumber\\ \end{eqnarray*}$$

Experimentally, we have measured the bound RGG fraction for each concentration of GQ. From the fitting of the cross-correlation functions into Eq. (7), we have experimentally determined the amplitude (*S*) and time scale (τ_S_) of the interaction dynamics. The time scale of this interaction dynamics, ${\tau _S}$, is the inverse of the sum of the association and dissociation rate, i.e

(12) $$\begin{equation*}{\tau _S} = \frac{1}{{{k_{on}}\left[ {GQ} \right] + {k_{off}}}}\; = \frac{1}{{{k_{on}}\left( {\left[ {GQ} \right] + K} \right)}}\;\end{equation*}$$

Combining the measurements of the bound fraction (Eq. 11) with the characteristic time ${\tau _S}$ (Eq. 12), we can unequivocally determine both the interaction rate constants *k*_on_ and *k*_off_, without making any extra hypothesis. Here, the value *K* for different GQ concentrations is determined using equation (11). Then, *k*_on_ is determined from equation 11 and finally, the dissociation rate constant, *k*_off_ is determined as

(13) $$\begin{equation*}{k_{off}} = \;K{k_{on}}\end{equation*}$$

Having determined *k*_on_ and *k*_off_, we can use this knowledge together with the measurement of the amplitude $S$ to determine the FRET efficiencies of the bound (*E*_1_) and unbound state (*E*_2_). We follow the method described by Levitus and coworkers.(57) The amplitude of the interaction dynamics is given by

(14) $$\begin{equation*}S\; = \frac{{{k_{off}}{k_{on}}{{\left( {{E_1} - {E_2}} \right)}^2}}}{{\left[ {{k_{on}}\left( {1 - {E_1}} \right) + {k_{off}}\left( {1 - {E_2}} \right)} \right]\left( {{k_{on}}{E_1} + {k_{off}}{E_2}} \right)}}\;\end{equation*}$$

To quantify the FRET efficiency, we make assume that *E*_2_$ \approx$ 0.02. This is verified by the overall global analysis of the data. The FRET efficiency (*E*_2_) of the unbound state would be extremely small as the complex is dissociated. However, this value would be never zero due to background signal and also due to the presence of ZMW which can still promote some weak FRET between GQ and RGG even when they are not bound to each other (58). Hence, there will be some residual value for *E*_2_. Using the 2% assumed value of *E*_2_, together with the experimentally determined value of *S*, *k*_on_ and *k*_off_, we determined the value of *E*_1_ from equation (14). Such determined values of *E*_1_ for all the samples is shown in Supplementary Figure S9. To validate our approach, we again recomputed the value of *S* from equation (14) using the known value of *k*_on_, *k*_off_, *E*_1_ and *E*_2_ and plotted for all the sample concentration and sample types in Supplementary Figure S9A. We found the recomputed value *S* converges very well to the value of S determined from FCS which suggests that our assumption and approach is reasonably valid here.

#### Ensemble fluorescence measurements

Ensemble fluorescence measurements are performed in a Tecan 10 M microplate reader. A nanoquant plate (Tecan 16 Flat Black) is used as sample chamber. In our measurement we are keeping the concentration of GQ-A546 concentration fixed at 1 μM and vary the concentration of RGG-A647 from 1 to 100 μM. We use the quenching of donor fluorescence to determine the titration plot of FRET efficiency as a function of RGG-A647 concentration. The excitation is carried out at 520 nm where Alexa 647 dye has minimum absorption and the emission is recorded from 555 nm to 800 nm wavelength range.

#### Circular dichroism measurements

UV-vis and electronic circular dichroism (ECD) spectra were measured on a JASCO J-815 spectrometer equipped with a JASCO Peltier cell holder PTC-423 to maintain the temperature at 20°C. A quartz photoelastic modulator set at l/4 retardation was used to modulate the handedness of the circular polarized light at 50 kHz. The CD spectrometer was purged with nitrogen during the recording of spectra. The UV absorption and ECD spectra were recorded simultaneously using the buffer as a reference.

## RESULTS AND DISCUSSIONS

### FCCS-FRET titration measurements in the ZMW

**Figure 1.**
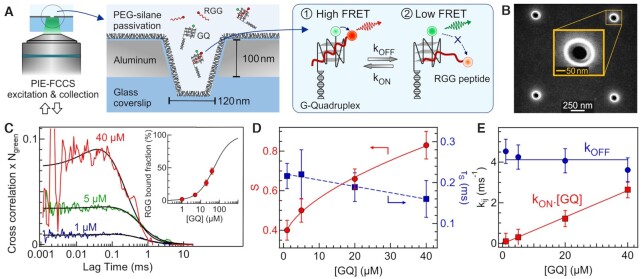
GQ–RGG interaction dynamics monitored at μM concentrations inside a ZMW nanoaperture. (**A**) Scheme of the experiment. *k*_on_ and *k*_off_ are the association and dissociation rate constants respectively. (**B**) Scanning electron microscopy image of a 120 nm ZMW nanoaperture. (**C**) FCCS curve at different GQs concentrations. The RGG concentration is fixed at 1 μM in all the measurements. Black lines are the FCCS fits according to equation 7. The inset shows the bound fraction of RGG peptide as a function of GQs concentration together with a Hill equation fit (Eq. 10) to determine the dissociation constant. (**D**) Amplitude S and time scale τ_S_ of the interaction dynamics as a function of GQs concentration. (**E**) Association *k*_on_.[GQ] and dissociation *k*_off_ rate as a function of GQs concentration.

Figure 1A represents the experimental scheme. We use a 10 mM Tris, 100 mM KCl, 2% Tween 20 buffer at pH 7.5 to determine the dissociation constant of the GQ–RGG complex. Circular dichroism spectroscopy data confirm that the GQ is folded at 100 mM KCl (Supplementary Figure S3, Supporting Information). For the FCCS titration, the RGG concentration is fixed at 1 μM while the GQ concentration is varied from 1 to 40 μM (Figure 1C). The observation volume for the 120 nm ZMW is measured to be 1.2 × 10^–18^ l (Supplementary Figure S4), which is 1000-fold smaller than the femtoliter confocal volume and stands in correct agreement with numerical simulations of the light propagation inside the nanoaperture (58). The evolution of the bound RGG fraction with respect to GQs concentrations (inset, Figure 1C) is fitted into a Hill equation (Eq. 10) to determine the dissociation constant *K*_D_, for this binding interaction. The *K*_D_ value determined from this fit is found to be 50 ± 2 μM indicating that at least 50 μM of GQs is required to bind 50% RGG to GQs. This value stands in good agreement with similar data determined from ensemble-based spectroscopy (25). This high *K*_D_ value confirms the need for ZMW in order to perform FCCS at such micromolar concentrations.

Remarkably, the FCCS curves exhibit an anticorrelated rise term in the sub-ms lag times which becomes more prominent at higher GQs concentrations. This anticorrelation comes from the Förster resonance energy transfer (FRET) between the Alexa Fluor 546 on the GQs and the Alexa Fluor 647 on the RGG peptide (Figure 1A) (53,56,57). Ensemble measurements confirm the quenching of the donor dye on the GQs in presence of the acceptor-labelled RGG peptide (Supplementary Figure S5). From the FCCS fits (Supplementary Figure S6), we extract the amplitude *S* and time scale τ_S_ of the GQ–RGG interaction dynamics (Figure 1D). S increases with the GQs concentration, while the time scale of the interaction dynamics remains more or less similar around 200 μs. The fast sub-ms timescale of the RGG–GQ dynamics observed in the present study was not known previously. Using the method described in section 2.2.5, we then extract the dissociation *k*_off_ and association rate *k*_on_.[GQ] of the interaction dynamics (Figure 1E). The association rate *k*_on_.[GQ] increases with the GQs concentration with *k*_on_ = 0.07 m s^–1^.μM^–1^ while *k*_off_ remains constant at 4 m s^–1^. Higher GQs concentrations increase the probability of association with RGG while the dissociation rate reflects the decay probability of the GQ–RGG complex which is independent of the GQs concentration. The evolution of the association and dissociation rate constants with the GQ concentration thus confirm the validity of our measurements. Note that conformational dynamics of the GQ–RGG complex can also induce a change in the FRET efficiency between Alexa 546 in GQ and Alexa 647 in RGG. We hypothesize that the association and dissociation of RGG from GQ occurs gradually. When RGG approaches GQ initially few contacts are formed between GQ and RGG before final binding. Similarly, during dissociation RGG detaches itself from GQ gradually, contributing to the FRET fluctuations. Therefore, the anticorrelation in the FCCS comprises both the conformational, association and dissociation kinetics between GQ and RGG.

### FCCS-FRET in the ZMW as a function of KCl concentration

**Figure 2.**
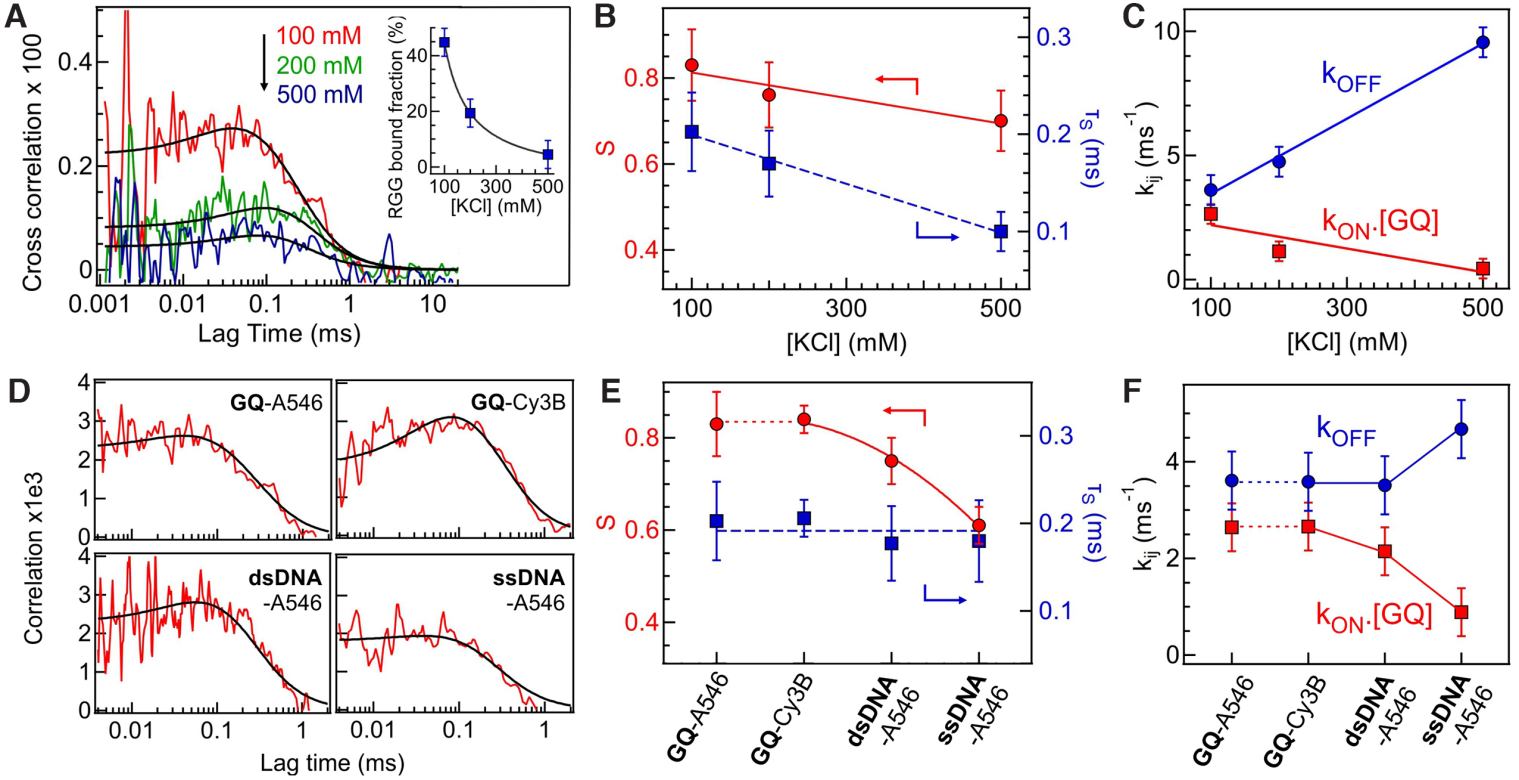
Influence of salt concentrations and DNA sequence. (**A**) FCCS curve at different KCl concentrations. The RGG and GQ concentrations are 1 and 40 μM, respectively. The inset shows the bound fraction of RGG peptide. (**B**) Amplitude *S* and characteristic time τ_S_ as a function of KCl concentration. (**C**) Association *k*_on_.[GQ] and dissociation *k*_off_ rate as a function of KCl concentration. (**D**) FCCS curves obtained for different DNA samples: Alexa 546 labelled G-quadruplex (GQ-A546), Cy3B labelled G-quadruplex (GQ-Cy3B), Alexa 546 labelled 51 bases DNA single strand (ssDNA-A546), and Alexa 546 labelled 51 bp DNA double strand (dsDNA-A546). (**E**) *S* and τ_S_ for the different DNA samples. (**F**) Association *k*_on_.[GQ] and dissociation *k*_off_ rates for different DNA samples.

**Figure 3.**
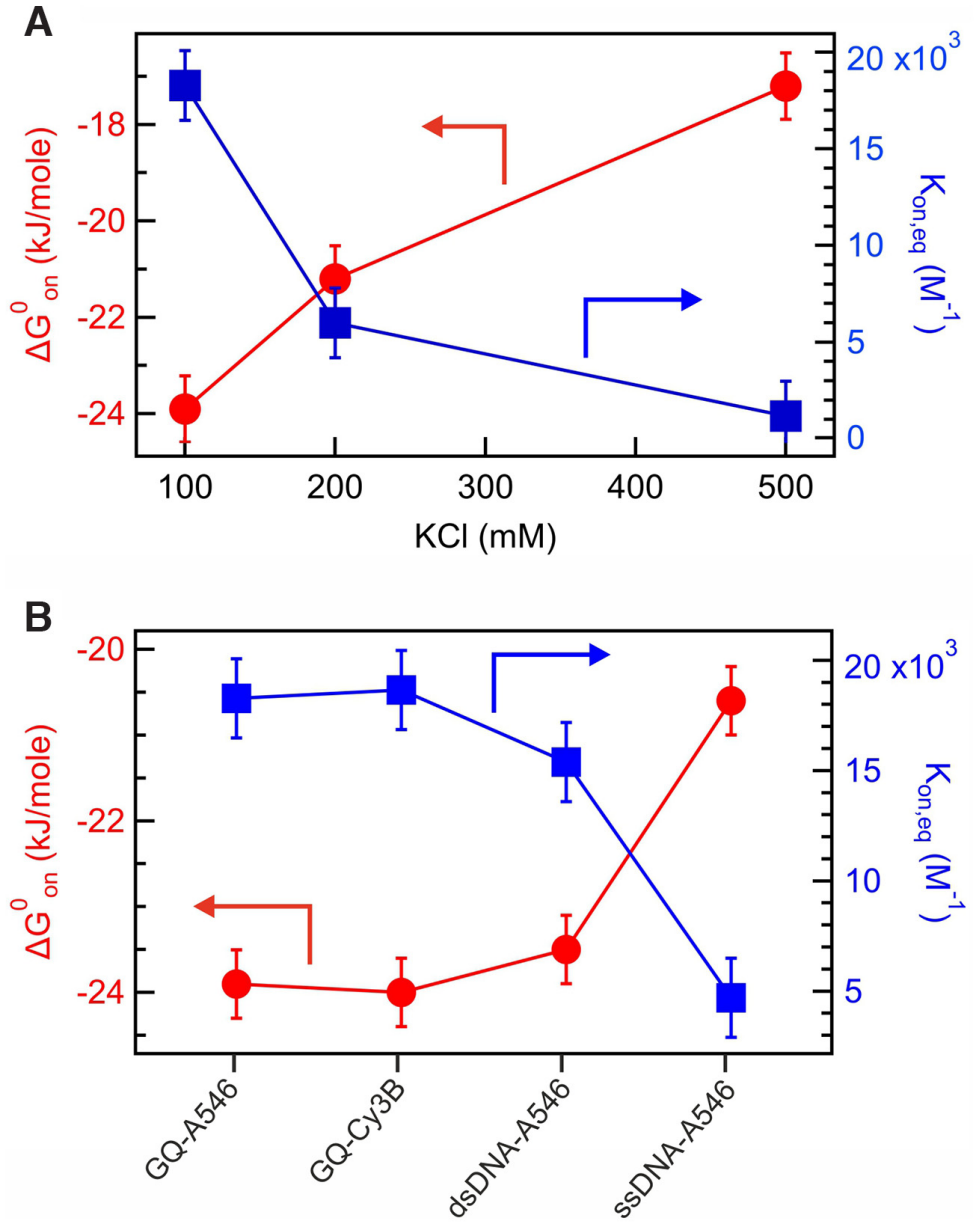
Thermodynamics of the RGG association. (**A**) Plot of equilibrium constant (*K*_on,eq_) and free energy change for association ($\Delta {{G}}_{{\rm{on}}}^0$) between GQs and RGG as a function of KCl concentration. (**B**) *K*_on,eq_ and $\Delta {{G}}_{{\rm{on}}}^0$ for the association of RGG with different DNA structures.

We next explore the electrostatics influence in the interaction between GQ and RGG. The net electric charge of GQ DNA and RGG peptide is –64 and +6.9 respectively. To gradually screen out the electrostatic charges, we perform FCCS-FRET measurements in the ZMW at increasing KCl salt concentrations from 100 to 500 mM. Additional data in the Supporting Information Supplementary Figure S10 show that similar results are obtained while replacing KCl by NaCl. Figures 2A–C and Supplementary Figure S7 report the KCl concentration influence. The GQ and RGG concentrations are fixed at 40 μM and 1 μM respectively. The FCCS amplitude and the RGG bound fraction decrease with increasing KCl concentrations (Figure 2A). Monitoring the sub-ms dynamics reveals that both the amplitude S and relaxation time τ_S_ decrease with increasing KCl concentrations (Figure 2B). The association *k*_on_.[GQ] and dissociation *k*_off_ rate constants show opposite behaviors and dependence with the salt concentration (Figure 2C). *k*_off_ strongly increases with KCl concentrations, while *k*_on_.[GQ] slightly decreases. The equilibrium constant for association (*K*_on,eq_) is determined from the ratio of *k*_on_ and k_off_, i.e. *K*_eq,on_ = *k*_on_/*k*_off_. Here, *K*_on,eq_ is the inverse of the equilibrium dissociation constant *K*_D_, i.e. *K*_on,eq_ = 1/*K*_D_. The free energy change ($\Delta {{G}}_{{\rm{on}}}^0$) for association can be determined from *K*_eq,on_ using the formula $\Delta {{G}}_{{\rm{on}}}^0$= –*RT*ln *K*_on,eq_. The *K*_eq,on_ value decreases with increasing KCl concentration (Figure 3A). The value of $\Delta {{G}}_{{\rm{on}}}^0$ also becomes less negative at higher KCl concentration (Figure 3A). This indicates that the association between GQ and RGG becomes thermodynamically less favourable with increasing KCl and screening charges. Altogether, our data clearly show that the GQ–RGG interaction is indeed largely controlled by electrostatics. High salt concentrations screen out the electrostatic interactions, reducing the binding affinity, increasing the dissociation rate and makes the association process thermodynamically unfavorable. While these trends were quite expected, Figure 2C importantly quantifies the influence of the KCl concentration on the interaction dynamics.

### Effect of fluorescent label and binding specificity of RGG to GQ

Figure 2D-F and Supplementary Figure S8 investigate the specificity between GQs and RGG and the influence of the fluorescent label. Four different DNA samples are considered: GQs labelled with either Alexa Fluor 546 or Cy3B, and two DNA sequences of 51 bases (single or double stranded) labelled with Alexa Fluor 546. All the measurements are conducted at 40 μM DNA and 1 μM RGG with 100 mM KCl. The bound fraction for GQs labelled with Alexa 546 or Cy3B are similar with 45 and 46% respectively. Likewise, the interaction dynamics amplitude and characteristic time are unchanged by the fluorescent dye (Figure 2E) leading to similar rates (Figure 2F). These data confirm that our observations do not depend on the choice of the fluorescent dye. Moreover, the bound fractions for the double and single stranded DNA decrease to 38% and 16% respectively. A marginal increase in the dissociation rate *k*_off_ is observed from 3.61 ms^–1^ in GQs to 4.68 ms^–1^ in single stranded (ss) DNA, while the association rate *k*_on_ decreases significantly from 0.07 μM^–1^ ms^–1^ in GQ to 0.05 μM^–1^ ms^–1^ in dsDNA to 0.02 μM^–1^ms^–1^ in ssDNA (Figure 2F). Almost 4 fold decrease in the equilibrium constant for association (*K*_on,eq_) is observed from GQs to ssDNA (Figure 3B). The $\Delta {{G}}_{{\rm{on}}}^0$ is also becoming less negative for other DNA structures in comparison to GQ (Figure 3B). All these results indicate that the association of RGG with other DNA structures becomes thermodynamically and kinetically less favorable as compared to GQs. The net electric charge of the RGG-peptide is +6.9, while GQ-A546(Cy3B), ssDNA-A546, and dsDNA-A546 have –64, –51 and –102 electric charge respectively. The difference between ssDNA and dsDNA can be explained by the twice higher negative charge of the dsDNA sample. However, despite GQs have lesser negative charge than the dsDNA, we still monitor a higher affinity for the GQ–RGG interaction with larger bound fraction and larger k_on_. This indicates some specific affinity between RGG and GQs which is not only driven by electrostatic interactions and may involve H bonding between arginine and the guanine bases of the GQs.(25,32,33)

## CONCLUSIONS

FCCS-FRET coupled with ZMWs provides a powerful tool to explore the kinetics and thermodynamics of biomolecular interactions with single-molecule resolution at μM concentrations. Investigating a regime inaccessible to diffraction-limited microscopes provides mechanistic insights into the RGG–GQ interaction by evidencing sub-ms dynamics and high μM dissociation constant of the GQ–RGG complex. Our approach allows to fully characterize the association and dissociation kinetics of the GQ–RGG interaction. Comparing the kinetics and thermodynamics of the RGG association and dissociation at different salt concentration and for different DNA types indicates unique information about the nature and specificity of this interaction. While a large part of the interaction is driven by electrostatics, a specific affinity between GQs and the RGG-rich peptide is still preserved. These insights are important for improving our understanding of GQs regulation mechanisms and using them as anticancer treatment. Overall, FCCS-FRET in the ZMW can be broadly applied to investigate a large library of peptides or proteins interacting with GQs at μM concentration. This method remains quite easy to implement on a time-resolved microscope and should stimulate future works exploring GQs with different loop lengths and topologies, as well as peptides with different mutations. Improving our knowledge about GQs-proteins interactions is important to develop the antiviral and anticancer drug therapy applications involving G-quadruplexes.

## ASSOCIATED CONTENT

### Supporting information

Raw HPLC and mass spectrometric data of the RGG peptide provided by the manufacturer (Supplementary Figure S1), demonstration of anticorrelation in the FCCS curve (Supplementary Figure S2), circular dichroism spectra at different KCl concentrations (Supplementary Figure S3), plot of number of molecules vs concentrations inside ZMW (Supplementary Figure S4), ensemble fluorescence spectra (Supplementary Figure S5), crosscorrelation, green and red autocorrelation curves obtained in the FCCS titrations measurements at different GQs concentrations (Supplementary Figure S6), fluorescence cross and autocorrelation curves obtained for different KCl concentrations (Supplementary Figure S7), Results of the FCCS measurements performed for different DNA sequence (Supplementary Figure S8), variation of the amplitude (*S*) of the interaction dynamics and FRET efficiency (*E*_1_) of the bound state as a function of GQ concentrations, KCl concentrations and different DNA types (Supplementary Figure S9), and comparison between NaCl and KCl (Supplementary Figure S10).

## DATA AVAILABILITY

All data are available from the corresponding author upon reasonable request.

## Supplementary Material

gkab1002_Supplemental_File
