# Supplementary material for: Fast interaction dynamics of G-quadruplex and RGG-rich peptides unveiled in zero-mode waveguides
Source: Nucleic Acids Res. 2021 Nov 17;49(21):12348–57. doi: 10.1093/nar/gkab1002 (PMC8643622; doi:10.1093/nar/gkab1002)
Supplement: gkab1002_Supplemental_File [file gkab1002_supplemental_file.pdf]

Supporting Information for

Fast Interaction Dynamics of G-Quadruplex and RGG-rich Peptides Unveiled in Zero-Mode Waveguides

Satyajit Patra, Jean-Benoît Claude, Jean-Valère Naubron, and Jérome Wenger*

Aix Marseille Univ, CNRS, Centrale Marseille, Institut Fresnel, 13013 Marseille, France.

* email : jerome.wenger@fresnel.fr

Contents

Supporting Figures

1. *Raw HPLC and mass spectrometric data of the RGG peptide (**Figure S1**)*
2. *Demonstration of anticorrelation in the FCCS curve (**Figure S2**)*
3. *Circular dichroism spectra at different KCl concentrations (**Figure S3**).*
4. *Evolution of number of molecules inside ZMW as a function of concentration* (***Figure S4***).
5. *Fluorescence spectra obtained from the ensemble fluorescence measurement (**Figure S5**).*
6. *Cross correlation, green autocorrelation and red autocorrelation curves obtained from the FCCS titration measurements (**Figure S6**).*
7. *Fluorescence cross and autocorrelation curves obtained from the measurements at different KCl concentrations (**Figure S7**).*
8. *Results of the FCCS measurements performed for different DNA sequence (**Figure S8**).*
9. *Variation of the amplitude of the interaction dynamics (S) and FRET efficiency of the bound state (E_1) as a function of GQ concentrations, KCl concentrations and different DNA (**Figure S9**).*
10. *Comparison between NaCl and KCl (**Figure S10**).*

1. Raw HPLC and mass spectrometric data of the RGG peptide

Data File: S:\MSD-11\2020\03-MAR\110320\ ->

Injection Date : Wed, 11. Mar. 2020
Sample Name : 210220XS06o|41774Pat 1b
Acq Operator : SYSTEM
Acq. Method : D:\CHEMSTATION\1\METHODS\5-95-6M M
column used :
Analysis Method : C:\Chem32\1\METHODS\DEF_LC.M

Sample Info : 41774_1b-Mr. Dr. Satyajit Patra|H-RGRGRGRGGGSGGGGRGRG-Cys(Alexa647)-NH2 ->

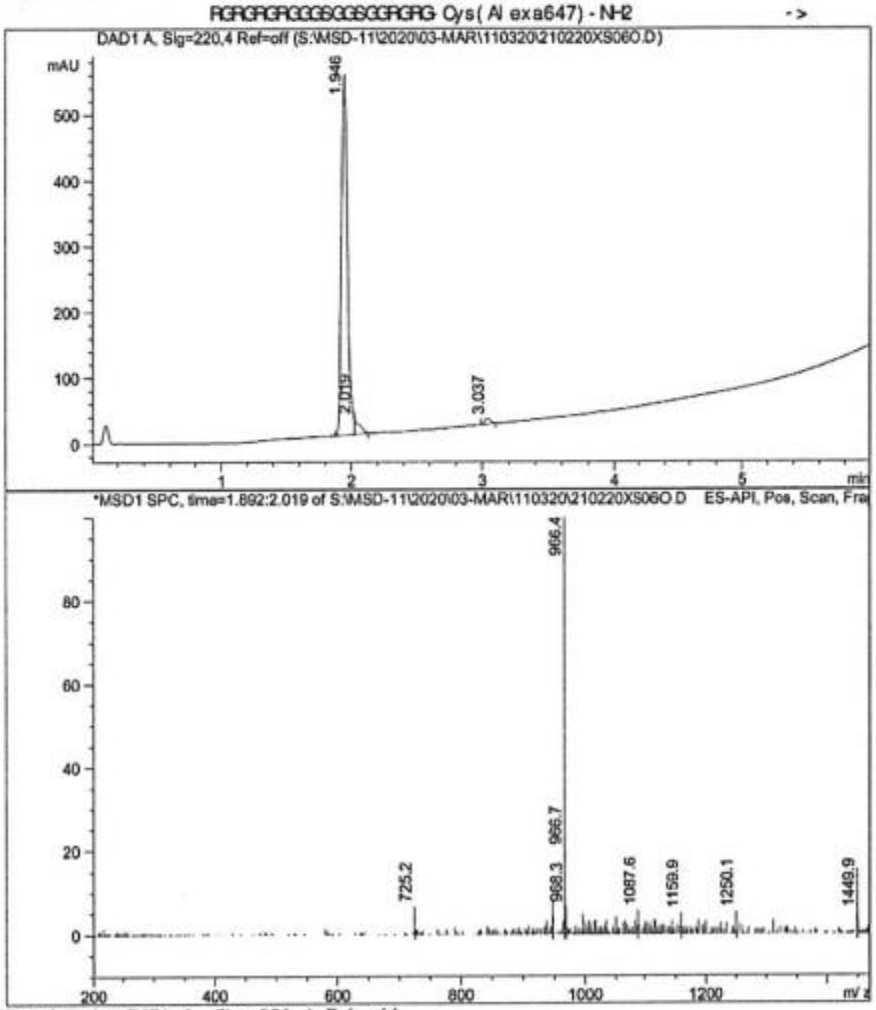

Signal 1: DAD1 A, Sig=220,4 Ref=off

Peak #	RT [min]	Width [min]	Area	Area %
1	1.946	0.1	1772.9	95.1
2	2.019	0.0	61.9	3.3
3	3.037	0.1	28.5	1.5

Figure S1: The raw HPLC and mass spectrometric data obtained for the RGG peptide. This data has been provided by the manufacturer. The purity of the sample is found to be 95.1%.

2. Demonstration of anticorrelation in the FCCS curve

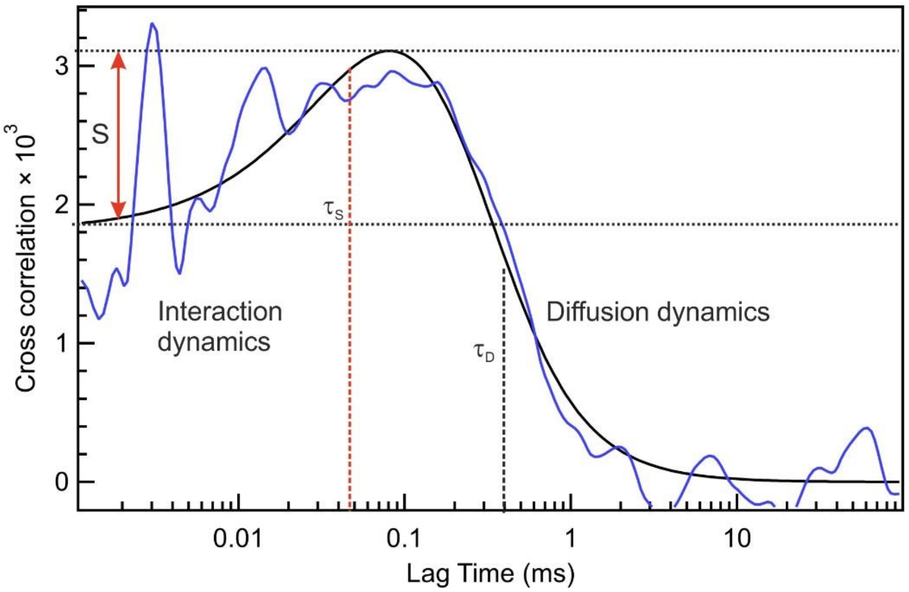

Figure S2: Demonstration of the interaction dynamics between GQ and RGG obtained from the fit to the cross correlation curves. S represents the amplitude of the interaction dynamics while τ_S is the time scale of the interaction dynamics. Here the blue trace is the FCCS data and black line represents fit to the curves according to equation 7.

3. **<u>Circular dichroism (CD) spectra at different KCl concentrations</u>**

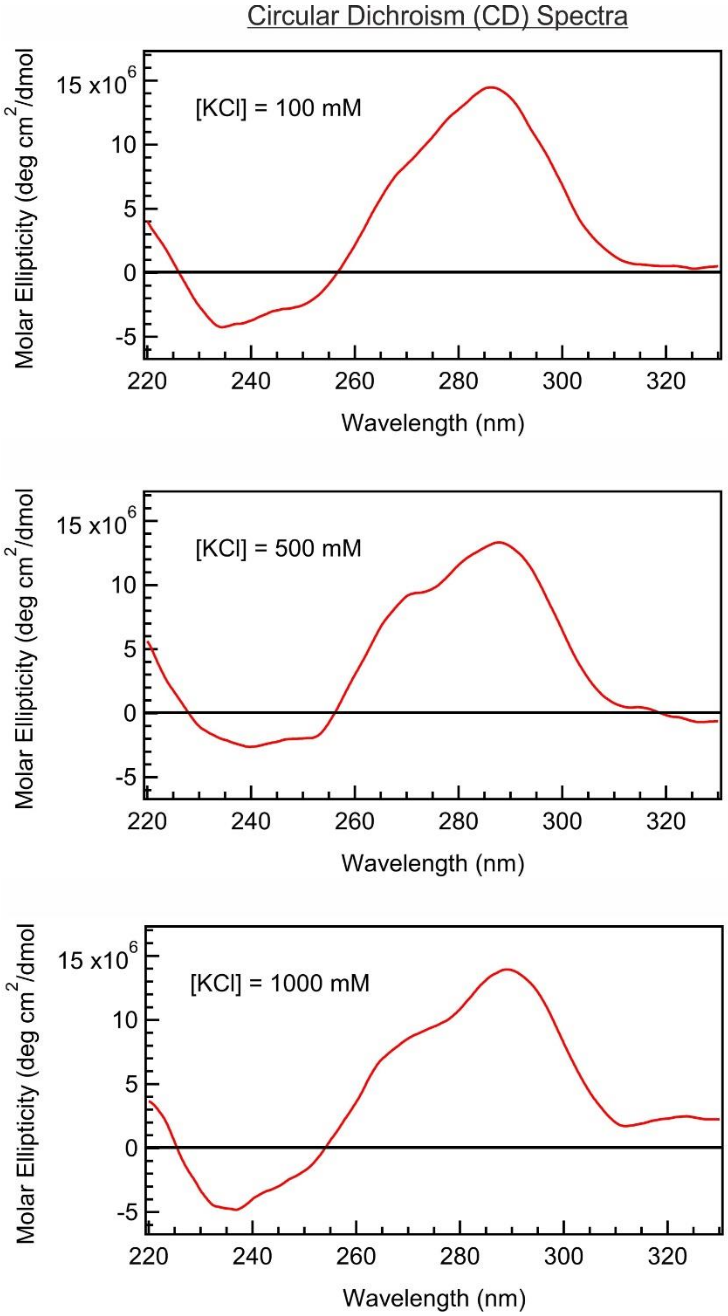

Figure S3: **Circular dichroism (CD) spectrum of the G-quadruplex (GQ) at 100 mM, 500 mM and 1000 mM KCl concentrations**. To obtain the CD spectrum of only GQs the CD signal of GQ-duplex stem is subtracted from CD signal of the duplex stem. The CD signal indicating the formation of folded GQ structures and it also suggests that the GQ exists in multiple conformations i.e a mixture of parallel, antiparallel and hybrid structures.(1)

4. Evolution of number of molecules inside ZMW as a function of concentration

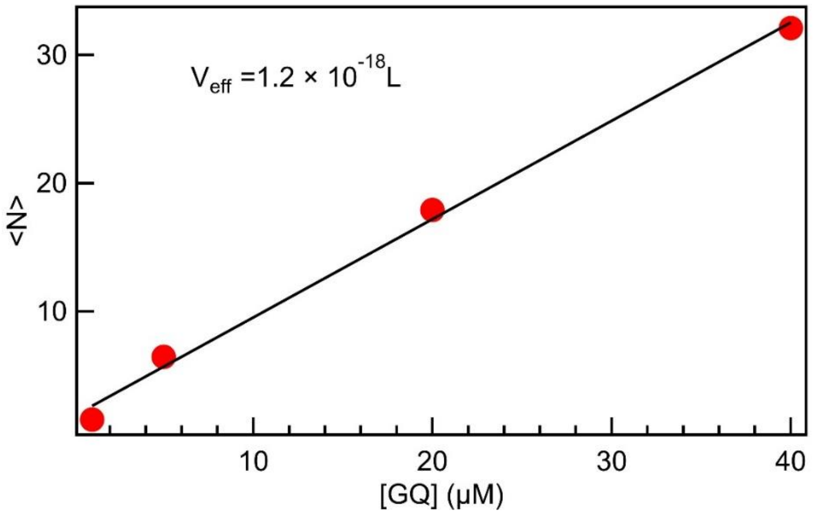

Figure S4: **Evolution of the number of molecules inside 120 nm ZMW as a function of GQ concentrations**. Here red symbol is the data and black line is the linear fit. The error bars are smaller than the size of the symbol. The effective observation volume (V_{eff}) is determined from the slope of the linear fit and is found to be 1.2 attoliter i.e 1.2 $\times$ 10^{-18} L. This data demonstrates that quantitative FCS measurements of the number of molecules are fully possible at micromolar concentrations using ZMW nanoapertures. The detection volume also matches the volume expected from numerical simulations.(2)

5. Fluorescence spectra obtained from the ensemble measurement

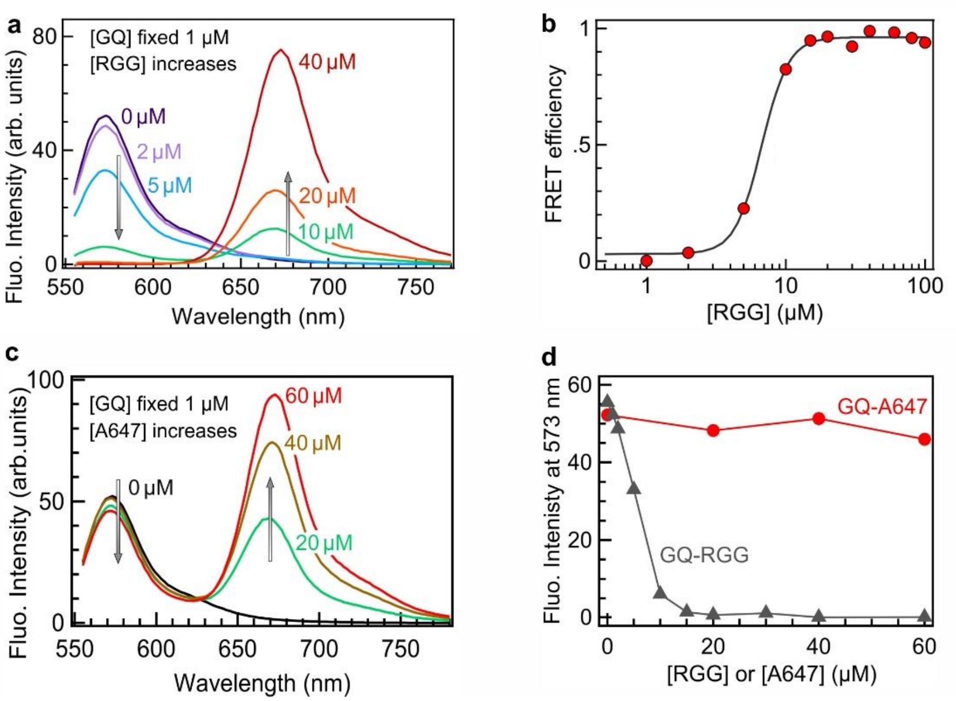

Figure S5: **Results of the ensemble FRET measurements**. (a) Ensemble fluorescence spectra recorded at 1 µM GQ and at different concentrations of RGG ranging from 2 µM to 40 µM. The donor fluorescence quenching at 570 nm by the acceptor has been used to compute the FRET efficiency. (b) Titration plot of FRET efficiency as a function of RGG concentrations. (c) Fluorescence spectra recorded at fixed 1 µM concentration of GQ while the concentrations of free Alexa 647 (A647) dye vary from 20 µM to 60 µM. This is a control to show that the donor quenching seen in (a) is not induced by the micromolar concentration of the free acceptor dye. Strong donor quenching requires the interaction between GQ and RGG peptide, and is a signature of FRET. (d) Comparison of the variation of the green emission peak intensity as a function of RGG and A647 concentrations respectively. Donor fluorescence does not quench even at a very high concentration of free A647 dye while donor emission almost completely quenches at high RGG concentrations, demonstrating the occurrence of FRET between Alexa dyes in the GQ-RGG complex.

6. <u>Cross correlation, green and red autocorrelation curves obtained in the FCCS titration measurements</u>

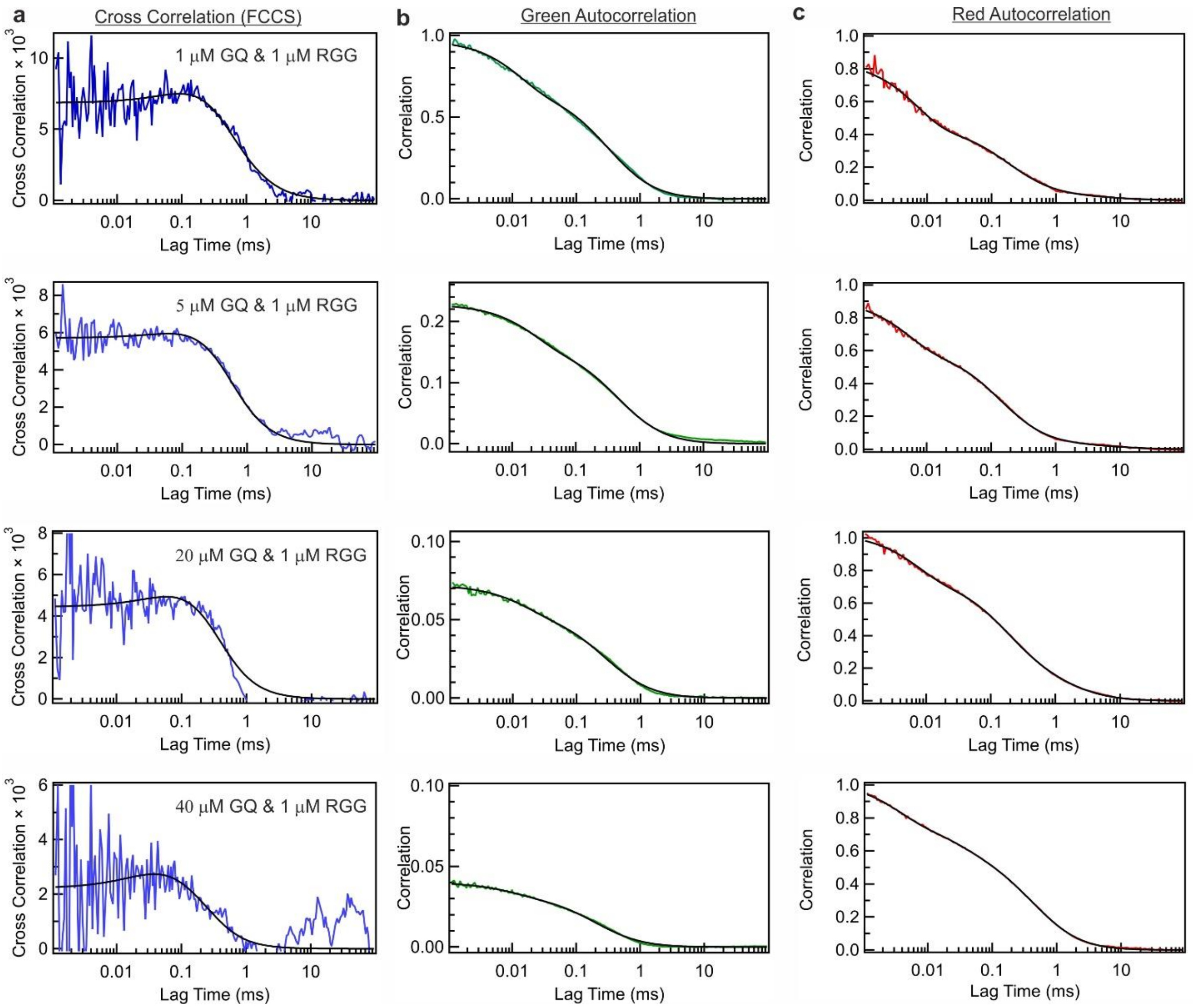

Figure S6: **Cross correlation, green and red autocorrelation curves obtained from the FCCS titration measurements**. Here, blue curves represent cross correlation curves, obtained from the cross correlation of the green and red fluorescence signals. During the titration the concentration of the RGG peptide is fixed at 1 μM concentration while the GQ concentrations vary from 1 μM, 5 μM, 20 μM to 40 μM. All the measurement is performed at 100 mM KCl concentration. Here, black curves represent the fit to the corresponding data.

7. <u>Cross correlation and auto correlation curves obtained from the experiments at different KCl concentrations</u>

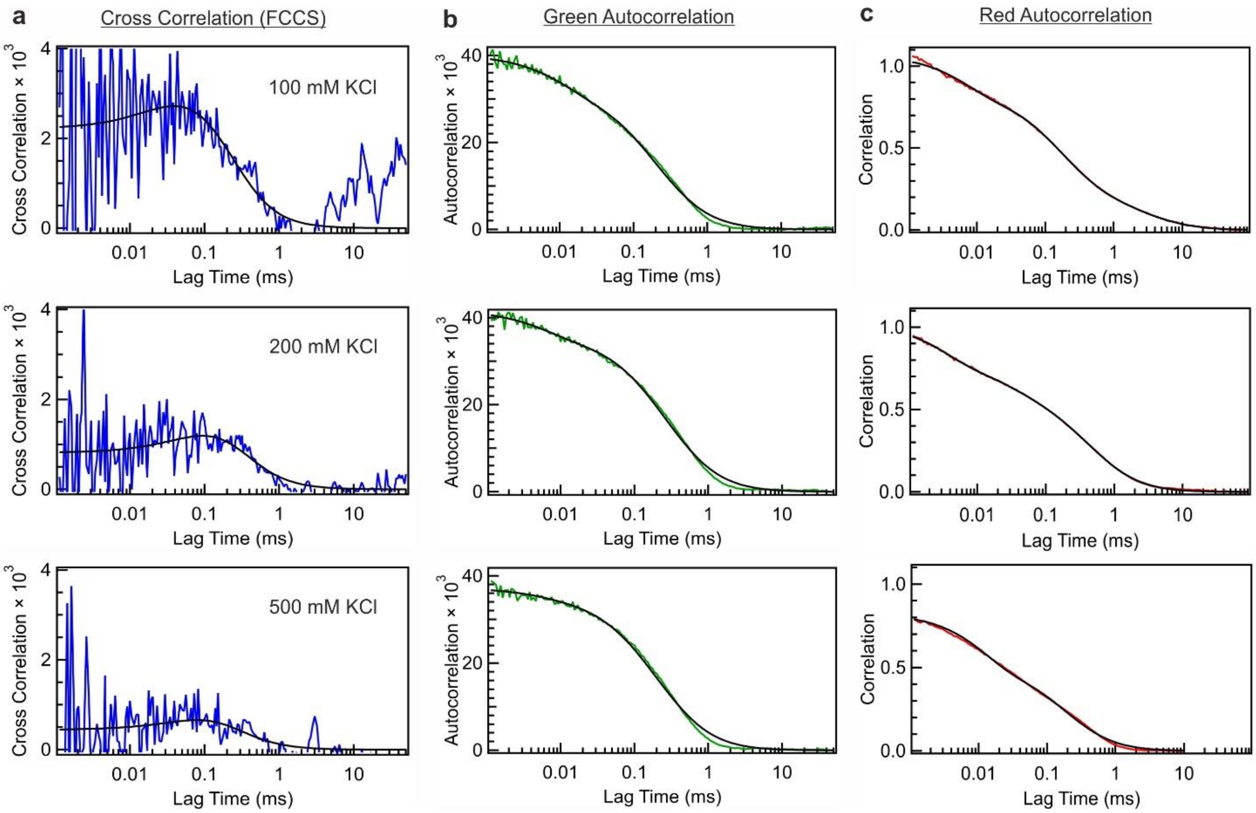

Figure S7: **Results of the FCCS measurements performed at different salt concentrations**. Cross Correlation curves are indicated by blue color while green and red color indicates the respective autocorrelation curves. In all the measurements GQ and RGG concentration is kept at 40 µM and 1 µM respectively. Black lines here represent the fit to respective data set.

8. Results of the FCCS measurements conducted for different DNA sequence

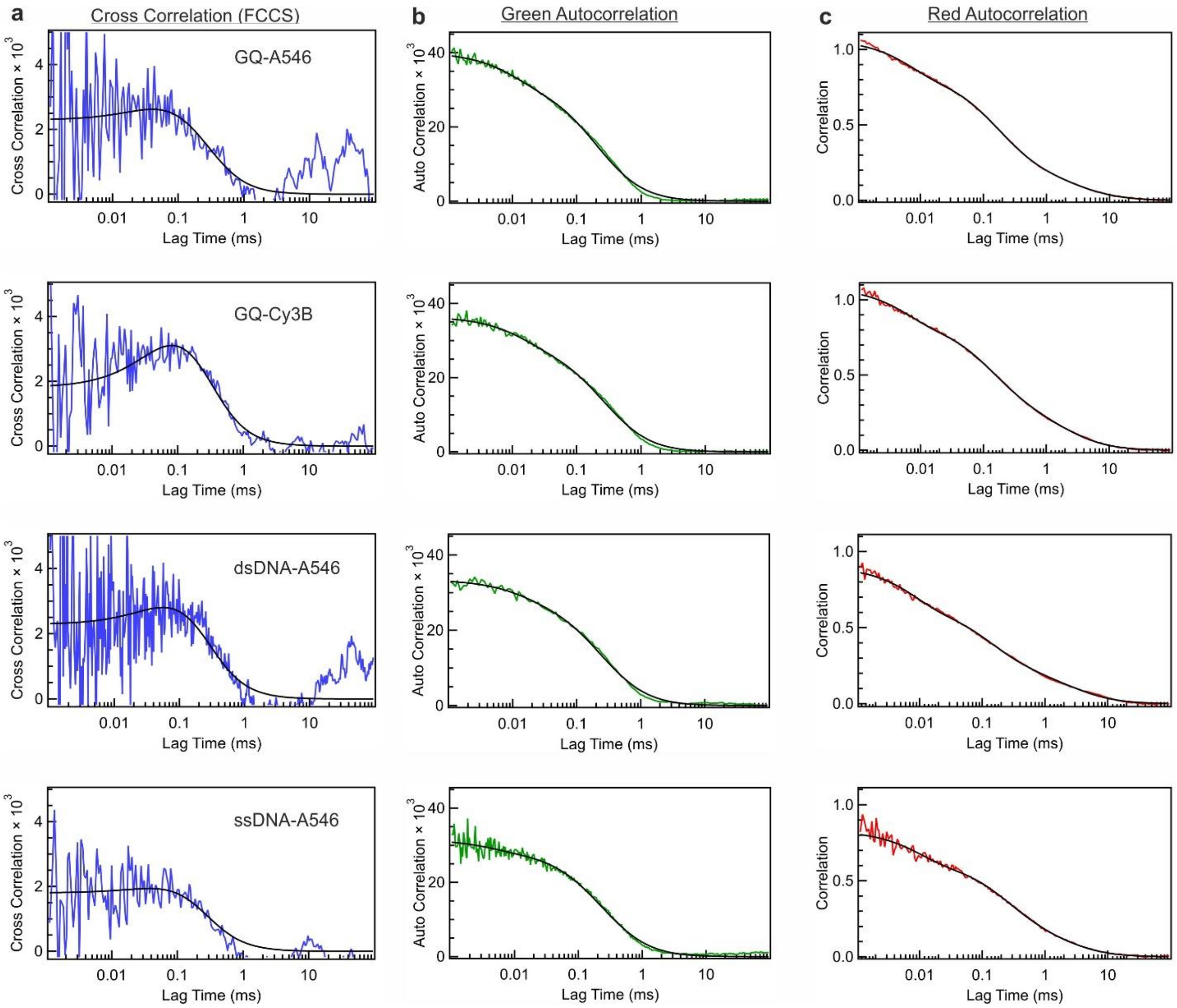

Figure S8: **Fluorescence cross correlation, green and red autocorrelation curves obtained from the FCCS measurements with different DNA**. Blue curves represent the cross correlation curves and green and red curves represent the corresponding autocorrelation curves. Here black line is the fit to the data. All the DNA concentration is fixed at 40 µM concentration. The RGG peptide concentration is kept at 1 µM. All the experiments are performed at 100 mM KCl concentration.

9. Variation of the amplitude of the interaction dynamics (S) and FRET efficiency of the bound state (E1) as a function of GQ concentrations, KCl concentrations and different DNA.

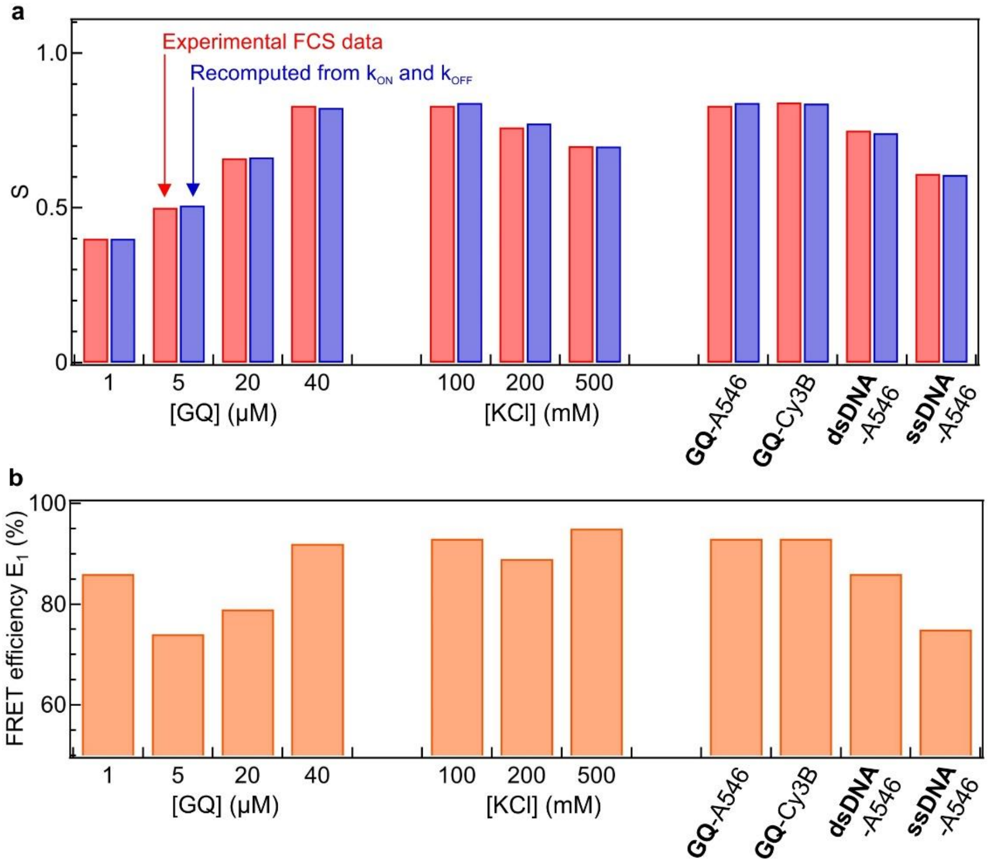

Figure S9: **Amplitude of the interaction dynamics (S) and FRET efficiency (E_1) of the GQ-RGG complex as a function of different sample concentrations and sample types**. (a) Plot of S determined from FCS and recomputed from the value of k_{on} and k_{off} as a function of GQ concentrations, KCl concentrations and different DNA samples. (b) The value of the FRET efficiency of the GQ-RGG complex (E_1) determined from equation 12 for all the measurements.

10. NaCl and KCl salts give similar results

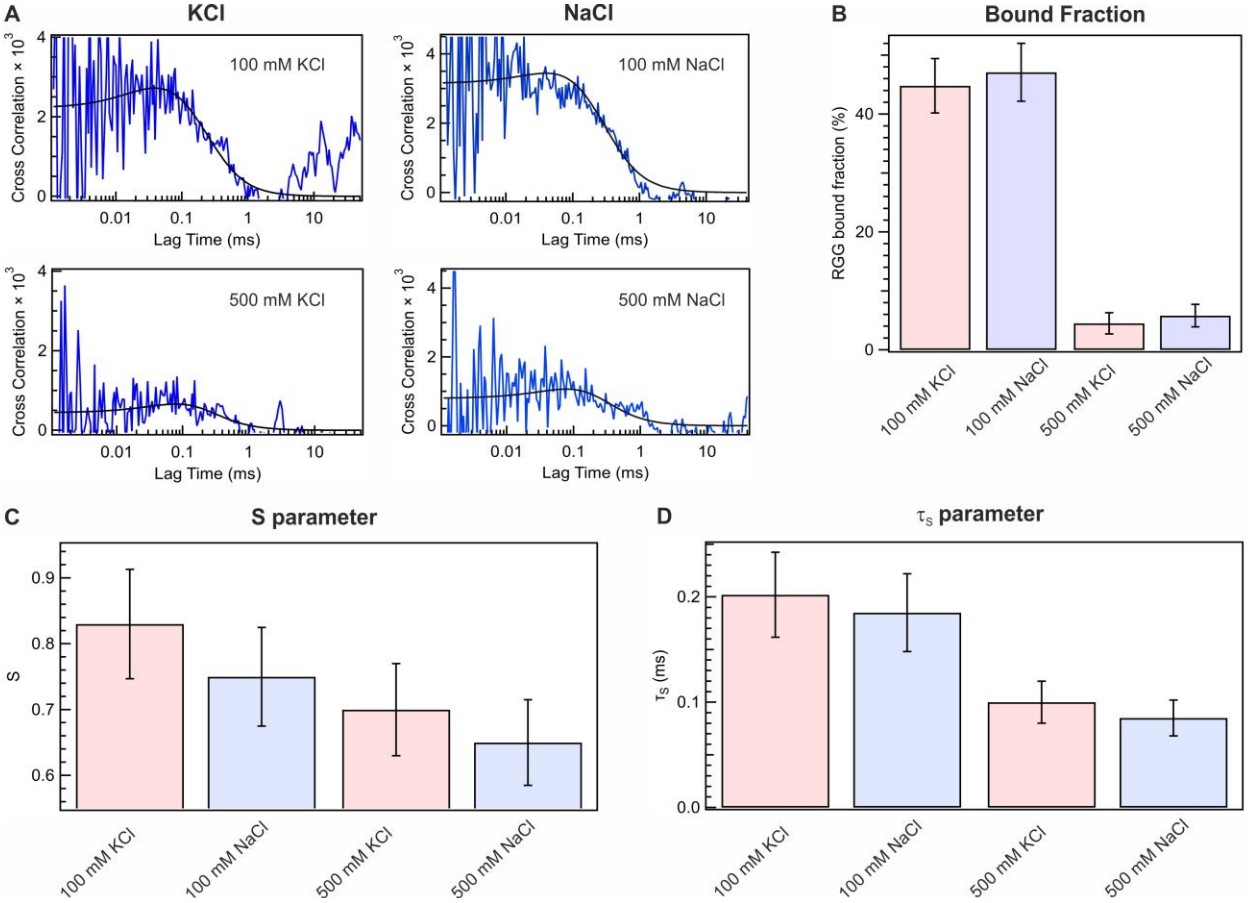

Figure S10: **Comparison between KCl and NaCl effect on the binding interaction between GQ and RGG.** (A) FCCS curves recorded at 100 and 500 mM KCl and NaCl concentrations. Here the concentrations of GQ and RGG are 40 µM and 1 µM respectively. (B) Plot of RGG bound fraction as a function of KCl and NaCl concentrations. (C) Amplitude of the interaction dynamics S and (D) time scale of the interaction dynamics as a function of KCl and NaCl concentrations.

Supplementary references:

1. del Villar-Guerra,R., Trent,J.O. and Chaires,J.B. (2018) G-Quadruplex Secondary Structure Obtained from Circular Dichroism Spectroscopy. *Angew. Chemie. Int. Ed.*, **57**, 7171–7175.

2. Punj,D., Ghenuche,P., Moparthi,S.B., de Torres,J., Grigoriev,V., Rigneault,H. and Wenger,J. (2014) Plasmonic antennas and zero-mode waveguides to enhance single molecule fluorescence detection and fluorescence correlation spectroscopy toward physiological concentrations. *Wiley Interdiscip. Rev. Nanomedicine Nanobiotechnology*, **6**, 268–282.
